# Lifetime Soy Intake and Adult Mammographic Density in Chinese Premenopausal Women

**DOI:** 10.3390/nu18071116

**Published:** 2026-03-31

**Authors:** Suzanne C. Ho, Norman F. Boyd, Wilson W. S. Tam, Winnie Yeo, Winnie C. W. Chu, Edwina K. F. So, Ruby Yu, Jean Woo

**Affiliations:** 1 JC School of Public Health and Primary Care, Faculty of Medicine, The Chinese University of Hong Kong, Hong Kong 999077, China; edwinaso@yahoo.com (E.K.F.S.); rubyyu@cuhk.edu.hk (R.Y.); 2 Princess Margaret Cancer Centre and Imaging Research, University of Toronto, Toronto, ON M5S 3G8, Canada; boyd@uhnres.utoronto.ca; 3 Alice Lee Centre for Nursing Studies, Yong Loo Lin School of Medicine, National University of Singapore, Singapore 119228, Singapore; nurtwsw@nus.edu.sg; 4 Department of Clinical Oncology, Faculty of Medicine, The Chinese University of Hong Kong, Hong Kong 999077, China; winnie@clo.cuhk.edu.hk; 5 Department of Imaging and Interventional Radiology, Faculty of Medicine, The Chinese University of Hong Kong, Hong Kong 999077, China; winniechu@cuhk.edu.hk; 6 Department of Medicine and Therapeutics, Faculty of Medicine, The Chinese University of Hong Kong, Hong Kong 999077, China; jeanwoowong@cuhk.edu.hk

**Keywords:** soy protein, mammographic density, life course exposure, Chinese premenopausal women

## Abstract

**Background:** Soy intake has been proposed as a protective factor for breast cancer, especially when exposure occurs early in life. Mammographic density (MD) is a strong predictor of breast cancer risk, but evidence linking soy intake at specific life stages to adult mammographic density remains limited. **Objective:** The objective of this study was to evaluate the associations between dietary soy intake at different life stages and MD in premenopausal Chinese women. **Methods:** Dietary soy intake was assessed using a validated soy food frequency questionnaire for the past 12 months and retrospectively for earlier life stages (childhood: 6–12 years; adolescence: 13–18 years; young adulthood: 20–34 years), with recall aided by a life history calendar. MD was measured from bilateral cranio-caudal mammograms using a standardized computer-assisted method. Multivariable regression models were used to evaluate associations between soy protein and isoflavone intake at different life stages and MD, adjusting for relevant confounders. **Results:** Among 815 premenopausal women (mean age 40.9 y), mean current soy protein and isoflavone intakes were 10.3 g/day and 22.0 mg/day, respectively. Soy intakes across life stages were moderately correlated (r = 0.33–0.81). After multivariable adjustment, soy protein intake during adolescence (β = −0.067, SE = 0.029, *p* = 0.023) and childhood (β = −0.071, SE = 0.032, *p* = 0.028) was significantly and inversely associated with adult MD. Young adult intake showed a non-significant inverse trend (β = −0.052, *p* = 0.075), and current intake showed no association (*p* = 0.93). Higher mean early-life (ages 6–18) and life course soy intakes were also inversely associated with MD (β range: −0.077 to −0.082; all *p* < 0.05). Women with consistently high early-life soy intake had 5.8–6.6% lower adjusted MD than those with consistently low intake. **Conclusions**: Early-life soy exposure may influence adult breast tissue composition and represents a potentially modifiable protective factor in breast cancer prevention. These findings carry important public health implications, particularly for populations experiencing dietary westernization.

## 1. Introduction

Diet is an important and potentially modifiable factor in cancer risk [1,2]. Soy foods have long attracted research interest because they contain isoflavones—bioactive compounds with estrogen-modulating properties that may influence hormonal pathways with potential cancer-risk-modifying effects [3,4]. Soy is a component of traditional Asian diets. Migrant studies have demonstrated that Asian Americans have substantially higher rates of hormonally dependent cancers, including breast and prostate cancer, compared with their counterparts living in Asia who retain traditional, isoflavone-rich soy diets [5,6,7].

Accumulating evidence suggests that not only the amount but also the timing of soy exposure may be important. Studies examining life course dietary patterns have shown that soy intake during childhood and adolescence may have a stronger influence on subsequent breast cancer risk than adult intake, potentially by shaping mammary gland development and modifying cancer susceptibility [8,9,10,11,12,13].

Mammographic density (MD)—the proportion of epithelial and stromal tissue relative to total breast area—is a well-established marker and a strong predictor of breast cancer risk [14,15,16,17,18]. MD reflects the cumulative influence of genetic, hormonal, reproductive, and environmental exposures across the life course [15,19,20,21]. Only a limited number of studies have evaluated the association between soy intake and MD, and findings remain inconclusive. Some observational studies have reported an inverse association between soy consumption and MD, whereas clinical trials—mostly conducted in low-soy-intake Western populations and involving short-term interventions—have generally shown little effect [22,23,24,25]. While higher lifetime soy intake has been associated with lower adult MD [26], emerging evidence indicates that early-life soy exposures may play a more critical role than adult intake in determining long-term breast tissue composition [9,10,13]. These observations are consistent with experimental models showing that pre-pubertal dietary and hormonal environments can influence mammary gland differentiation [13].

The Hong Kong population provides a unique setting in which to examine the relationship between life course soy intake and breast cancer risk. In recent decades, Hong Kong has undergone substantial dietary westernization, resulting in wide variability in soy intake. During this same period, breast cancer incidence has risen sharply, with lifetime risk increasing from 1 in 23 in 2000 to 1 in 13 in 2023 [27]. This dietary transition offers an opportunity to understand how variations in soy consumption across multiple life stages relate to MD in a population undergoing rapid dietary change.

This study aimed to examine the association between dietary soy intake during different life stages—including childhood and adolescence, young adulthood, recent intake, as well as cumulative intake—and adult MD in Chinese premenopausal women aged 35 to 45 years.

## 2. Materials and Methods

### 2.1. Study Population

The study population consisted of 815 healthy women aged 35–45 years recruited from the community. The sample size estimation was based on the planned use of multiple regression analysis. With eight independent variables expected in the final model and a small effect size anticipated, and according to Cohen’s guidelines [28], a minimum of 757 participants is required to achieve 80% statistical power at a two-sided significance level of 0.05. To account for an estimated 5% of incomplete responses, the target sample size was increased to 800 participants.

Stratified-cluster sampling method was used to select the housing estates in the Shatin district of Hong Kong according to the distribution of 3 housing types (public, private and home ownership) in the ratio of 3:5:2 [29]. Method of recruitment included door-to-door visits as well as written invitations placed in mailboxes of residents of the selected estates. Advertisements were also placed in the estate/district newspapers for subject recruitment to the study project. Women who responded to the invitation were screened for eligibility. An eligible subject was defined as a Chinese woman aged 35–45 years, pre-menopausal and without any of these conditions: history of benign breast disease or any cancer; menopausal (including surgical menopausal); use of exogenous estrogens in the past 12 months; on long-term use of antibiotics; on special diet or with a previous history of malabsorption disorders. To increase intake variations among the participants, subjects also responded to a brief questionnaire on the average intake of soy products per month/week, so that about one-third of the subjects would belong to the high (≥5/week), medium (3–4/week), or low (≤3/month) intake categories. After initial screening for eligibility, eligible women were invited to the Prince of Wales Hospital (a regional teaching hospital) for face-to-face interviews and anthropometric measurements by trained interviewers during October 2004–October 2006. Out of 9166 household visits, 541 eligible women joined the study with a response rate of 79.1% (541/684). A further 52 eligible women joined by responding to invitation letters, 279 from advertisements, and 22 through referral from participants. A total of 894 women joined the study. The distribution of the participants according to the housing types was 32.1% public, 48.2% private and 19.7% home ownership scheme, and these are in line with the intended recruitment ratio of 3:5:2.

The study received approval from the Joint Chinese University of Hong Kong-New Territories East Cluster Clinical Research Ethics Committee. All women signed the written informed consent form before being enrolled in the study.

### 2.2. Data Collection

On the initial visit, information on social demographic characteristics, family history of breast cancer, reproductive and lifestyle factors, overall dietary intake and soy food intake over the past year was collected based on a standardized questionnaire through face-to-face interview. Anthropometric measurements, including height, weight and waist circumference, were obtained based on a standardized protocol. A further visit was scheduled for assessment of soy intake during earlier life stages. Arrangement was also made for mammographic imaging. Assessors of dietary variables and covariates were blinded to the mammographic readings.

### 2.3. Assessment of Mammographic Density (MD)

Mammograms of cranio-caudal view of both breasts were taken at the Department of Radiology and Organ Imaging, Prince of Wales Hospital in Hong Kong, with dedicated mammographic equipment (Senographe DMR; General Electric Medical Systems, Milwaukee, WI, USA) using computed radiography. Mammographic images were digitized using VIDAR’s DiagnosticPRO Advantage film digitizer at a resolution of 300 dots per inch. The digitizer provided a 12-bit grey-level output that was linear in the range of 0–4.0 optical density. Each of the scanned images of the left and right breasts was assessed for the fraction of the breast occupied by fibroglandular tissue [16] by a computer-assisted method using Cumulus4 software (Version 4) developed by the University of Toronto [30]. The average of the left and right breast readings was used for analysis. All readings were conducted by a single independent assessor (N Boyd) who was blinded to the subject identity and the other study measurements. The films were first randomized and read in sets of 90. The reader first outlined the entire breast using an outlining tool on the computer screen; the computer counts the total number of pixels in the breast, representing the total breast area (cm^2^). The reader then drew a region of interest (ROI) to exclude the pectoralis muscle and other artifacts. Next, the reader used a tinting tool to color the areas within the ROI considered to contain the fibro glandular tissue. The software counts the number of tinted pixels within the ROI, representing the area (cm^2^) with high density. The MD is the area of dense tissue divided by the entire projected area of the breast and multiplied by 100. A random sample of 10% of the images was reread within and between each set to assess reproducibility. The intraclass correlation values were 0.93 and 0.86 for within and between sets respectively.

### 2.4. Assessment of Dietary Soy Intake

Overall dietary intake of the past 12 months was assessed based on a food frequency questionnaire. The assessment of dietary soy intake was based on a soy food frequency questionnaire (SFFQ) consisting of 32 soy food products. The SFFQ had previously been validated against 3217 dietary recalls among 145 Hong Kong Chinese midlife women with r = 0.53 (*p* < 0.0001) and r = 0.68 (*p* < 0.0001) respectively for validity and reproducibility [31]. Participants were asked to report their usual frequency of consumption and the average amount of daily intake of each food item over the past year. Assessment was aided by photographs of each food item with usual intake portion sizes and models of household containers.

Daily soy isoflavone intake was derived from the sum of three individual isoflavones (daidzein, genistein, and glycitein) and the total intake across all food items in the SFFQ. The estimation of soy isoflavone content was based on the Chinese University of Hong Kong Soy Isoflavone Database, which was generated from analysis of Hong Kong soy food items [32]. Soy protein and dietary energy intakes were derived from the Chinese Food Composition Tables 2002 and published data on local foods [33,34].

### 2.5. Assessment of Soy Food Intake at Earlier Life Stages (T_1_, T_2_, T_3_)

After the first visit with an interview on social demographic and lifestyle information and dietary intake over the past 12 months (T_0_), a second visit was scheduled for collection of information on soy food intake during the earlier life stages: early adulthood (20–34 years) (T_1_), adolescence (13–18 years) (T_2_) and childhood (6–12 years) (T_3_). To aid their recall, a life history calendar (LHC) with cue questions on key events and usual eating habits during those periods of life was given to the participants at the end of the first visit. They were asked to bring along answers to the cue questions contained in the LHC for their second visit. The cue questions were grouped into 3 domains and included: (i) places of residence, school or work, place for marketing, their usual places and common foods eaten for breakfast, lunch and dinner; (ii) their food preferences, usual soy foods/dishes consumed at home or outside and frequency of soy foods consumed per week; and (iii) who was their usual food preparer.

At the beginning of the second visit, each participant was asked to spend about 20 min going through the LHC, which was accompanied by audio and visual cues (popular songs, movie titles, photos and newspaper clippings of some landmark events in the 1970–90s). The interviewer then went through each of the LHC domains and the cue questions, starting from T_1_ (aged 20–34). After finishing a particular stage of the LHC, the interviewer administered SFFQ for that particular period with the use of soy food photos and frequently referring to cues from the LHC. Similar techniques were applied for recall of soy intake during T_2_ and T_3_ using SFFQ. The frequency of intake of each soy food item was assessed.

To assess the validity of participants’ recall of soy food intake in early life, separate interviews were conducted for participants’ food preparers (*N* = 76) [mothers (89%), sisters (11%)]. They were asked to recall the soy food consumption of the study participant during their adolescent years, based on the SFFQ. The LHC technique was also applied to assist their recall. The correlation of the log-transformed amount of soy food consumption reported by the participant and the food preparer was r = 0.428 (*p* = 0.004).

### 2.6. Statistical Analysis

Data for 816 subjects with information on mammographic analysis and on dietary soy intake during the four stages of life course were included for analysis. One subject reporting dietary energy intake over 4000 kcal/day was excluded from analysis. Descriptive statistics were generated for the basic characteristics of the study population. Categorical data were presented in the form of percentage distribution, and continuous data in terms of mean and standard deviation (SD). Amount of soy protein and isoflavone intake at different periods of life was described in terms of the overall amount, as well as by distribution in quartiles among the respondents.

Soy intakes at earlier life stages during childhood (6–12 y) (T_3_), adolescence (13–18 y) (T_2_), and young adulthood (20–34 y) (T_1_), as well as current intake (based on past year) (T_0_), were the independent variables. As only frequency of soy intake was assessed for the intakes during T_1_ to T_3_, the portion sizes for the different soy items indicated for the current intake (T_0_) were used to estimate the amount of soy intake consumed during the earlier life stages.

MD was the dependent variable. Multiple regression analyses adjusted for the potential confounders were conducted for the association between soy intake at different life stages and current MD. The potential confounders were selected based on a literature review on predictors of MD and breast cancer risks. These included age, marital status (married or cohabitation/never married/others), work status (homemakers/part time or volunteer work/full time work), height (m), weight (kg), waist circumference (cm), body mass index (weight in kg divided by square of height in meters), reproductive factors (age at menarche, age at first birth, number of live births, months of breast feeding), and physical activity index (based on modified Baecke questionnaire) [35]. Dietary energy and fat intake were derived from intake over the past 12 months. A core model on the association of soy intake at different life stages with MD was initially built based on analysis of variance on the selection of confounding variables (with *p*-value < 0.1) for multivariable analyses. The selected confounding variables were entered into multiple regression, and variance inflation factors were used to check for collinearity among the confounders. With the core model built, soy intake across different life stages was then individually added into the core model to examine its relationship with MD. Multiple linear regression analysis was performed comparing the adjusted mean MD values among participants with different levels of soy intake. Classification of levels of intake (high, moderate, low) was based on a scoring system summing the quartile intake category the participants belonged to at different life stages (Q1 = 1, Q2 = 2, Q3 = 3, Q4 = 4). SPSS version 26.0 (SPSS Inc., Chicago, IL, USA) was used for the analyses.

## 3. Results

This study examined the associations of recalled dietary soy intake through different life stages with MD in premenopausal women aged 35–45 y. The mean age of the participants was 40.9 y (SD, 2.94) (range 35–45 y). The majority were married, and about 90% had attained some secondary level of education. The proportions of women reporting current alcohol drinking (5.7%) and smoking (4.2%) were low. The mean BMI value was 23.1, and mean age at menarche was 13.0 y. The mean age at first birth was 27.2 y, and the mean number of live births among the parous women was 1.85. The mean current MD value was 38.8% (SD, 9.86) (Table 1). Table 1 also shows the distribution of frequency of soy food intake per week among participants, showing sufficient variation in intake in the study population.

The mean current (T) soy protein intake among the participants was 10.25 g/d (SD, 9.99) and isoflavone intake 22.0 mg/d (SD, 21.29), while the overall mean intakes for the four life stages were 10.85 g/d and 24.08 mg/d for soy protein and isoflavones respectively (Table 2). Significant correlations (*p* < 0.001) ranging from 0.327 to 0.799 and 0.324 to 0.807 among the soy protein or isoflavone intakes during the different life stages were noted, with higher correlations between the intakes at adjacent life stages (Table 3).

Regression of soy protein intake at different life stages on MD, after adjusting for the selected confounders (age, waist circumference, height, number of live births, and physical activity index), showed that intakes during T_2_ and T_3_ were significantly and inversely associated with MD (*p* < 0.05). Soy protein intake during T_1_ was inversely associated with MD but did not reach a statistical level of significance. However, no association between current soy protein intake (T_0_) and MD was noted. The mean of soy protein intake during the earlier life stages (T_2_ and T_3_) and through the life course (T_0_ to T_3_) showed a significant inverse association with MD (*p* < 0.05). Similar findings were observed for the association between soy isoflavone intake through the different life stages and MD (Table 4). Multivariate GLM analyses showed that women with consistently high intake (belonging to the high dietary intake quartiles) at or through the earlier life stages (T_1_ through T_3_) had significantly lower adjusted mean MD values compared with those with consistently low intake (Table 5a). Analyses of the relationship between consistently high or low soy isoflavone intake and MD showed similar results (Table 5b). We observed about 6% lower MD values among women with consistently high intake compared with those with consistently low intake during the earlier life stages (T_1_ to T_3_). However, a similar analysis conducted by including current soy intake did not show significant differences among the mean MD values.

## 4. Discussion

In this study, recent soy intake was not associated with adult mammographic density (MD); however, soy consumption during childhood and adolescence demonstrated a significant inverse association with MD. Women with consistently high early-life soy intake exhibited approximately 6% lower MD, a difference that is potentially meaningful for breast cancer prevention, given the well-established predictive value of MD for subsequent breast cancer risk [15,36,37,38,39]. Reductions of a similar magnitude have been reported in relation to adolescent stature [40], the menopausal transition [41], hormone therapy [42], and tamoxifen use [43], highlighting the potential biological significance of the observed effect.

Findings from previous studies examining soy intake and MD have been heterogeneous. While some observational studies have reported inverse associations, others have found null results [44,45,46]. Randomized controlled trials—predominantly conducted in populations with low habitual soy intake—have generally demonstrated minimal effects of isoflavone supplementation on MD [24,25,47,48,49]. Differences in habitual dietary patterns, intervention duration, and, critically, the timing of exposure likely contribute to these discrepant findings.

The timing of exposure appears particularly important for understanding soy’s biological effects [13,50,51]. Childhood and adolescence represent key developmental windows during which the mammary gland undergoes rapid structural and hormonal changes [51,52]. Consistent with this developmental framework, our findings align with the emerging data suggesting that soy intake during early life, rather than adulthood, may exert long-term protective effects on breast tissue. Maskarinec et al. observed inverse associations between childhood soy intake and adult MD [53], and similar relationships have been reported for other early-life exposures [8,9,10,11,12].

Biological plausibility is supported by mechanistic evidence that early-life soy intake can influence mammary development through estrogen-related pathways. Isoflavones preferentially bind Estrogen Receptor Beta (ERβ), promoting epithelial differentiation and counterbalancing ERα-mediated proliferative signaling [51,54,55,56]. These actions also modulate apoptosis, oxidative stress responses, and cell cycle regulation, processes relevant to long-term breast tissue composition.

Experimental data provide further support. Rodent models show that developmental isoflavone exposure enhances ductal branching, alters terminal end bud activity, and increases differentiated lobular–alveolar structures, accompanied by changes in maturation-related gene expression [54,55]. Non-human primate studies similarly demonstrate that pubertal soy intake modifies ER expression patterns across developmental stages, including reductions in ERβ before menarche and decreased ERα and GREB1 expression afterward [56]. Early-life genistein exposure may also induce epigenetic programming, providing a plausible mechanism for persistent modifications in mammary differentiation and hormonal responsiveness.

Overall, early activation of ERβ-dependent differentiation and attenuation of ERα-driven proliferation during hormonally sensitive windows may produce a more differentiated, less carcinogen-susceptible mammary architecture, aligning with epidemiologic evidence of lower mammographic density and reduced breast cancer risk among individuals with early-life soy exposure.

Lifestyle and anthropometric characteristics are also well-established factors associated with breast cancer risk [3]. For example, obesity is a pro-inflammatory state and has been identified as an important determinant of MD and breast cancer risk, with a recent study showing that the association between diet-related inflammation and MD is largely mediated through adiposity [57]. Detailed information on these factors was collected and evaluated as potential confounders, and the selected variables were included for adjustment when examining the association between soy intake and MD. In our cohort of premenopausal women, the majority had a body mass index within the normal range [mean 23.1 (SD 3.32)], and the prevalence of smoking and alcohol use was minimal, at 4.2% and 5.7% respectively. Consequently, age, height, waist circumference, number of livebirths and physical activity were included in the multivariable adjustment. Nonetheless, the possibility of residual confounding cannot be entirely excluded.

In summary, our results seem to align with the converging evidence from epidemiologic, experimental, and mechanistic studies suggesting that soy intake during childhood and adolescence may influence mammary gland maturation in ways that persist into adulthood, contributing to lower MD. Further research is needed to establish causality, identify optimal dose–timing parameters, and elucidate the underlying molecular pathways.

## 5. Limitations

There are several limitations in our study. Dietary soy intake was assessed retrospectively using an SFFQ to capture consumption from two to three decades earlier. Although such retrospective assessment is subject to recall error, similar approaches have been successfully employed in large epidemiologic studies evaluating adolescent and early-life diet [58,59], and have demonstrated reasonable reproducibility for distinct past periods such as adolescence [58,60,61]. For example, the mean nutrient correlation between mothers’ and participants’ own reports was 0.40 (range: 0.13–0.59) [58], with even higher correlations observed for specific foods such as dietary animal fat intake [59]. Additional evidence suggests that FFQs remain suitable for assessing adolescent diet when the recall period does not exceed 35 years [62] and are appropriate for use among premenopausal women [59].

In our study, recall was supported through the use of audiovisual aids and a life history calendar to enhance memory. Validation using reports from food preparers (*n* = 76) showed a correlation of r = 0.43 (*p* = 0.004) between participants’ recalled adolescent soy intake and preparers’ reports, consistent with previous mother–daughter dietary recall studies [59]. Participants were also unaware of their mammographic density (MD) measurements at the time of the interview, reducing the likelihood of differential recall bias. Nonetheless, because soy exposure data relied on recall of the distant past, recall bias and measurement error cannot be fully excluded and may limit the strength of inference.

In our analysis, soy intake levels were categorized into quartiles based on the population distribution. The absence of a true non-consumer or minimal-consumer comparison group may limit the interpretation of causality and effect size. Another limitation is the lack of biomarkers of soy exposure for validation. However, a recent study from Singapore reported moderate correlations between soy protein intake assessed by FFQ and urinary isoflavones (r = 0.46), supporting the FFQ’s ability to rank individuals by soy exposure [63]. Approximately 30–40% of Chinese women are equol producers capable of converting daidzein to the biologically active metabolite S-(Y)-equol [64]; equol-producing status was not assessed in this study and thus could not be examined as a potential effect modifier. Additionally, biochemical measures, such as hormonal and lipid profiles, were not available due to resource constraints, although their inclusion would have strengthened the interpretation of results.

Finally, although detailed sociodemographic, lifestyle, medical, and reproductive data were collected, permitting careful adjustment for potential confounders, residual confounding due to measurement error or unmeasured variables cannot be excluded and may have attenuated the observed associations.

## 6. Strengths of This Study

This study also has several notable strengths. Unlike studies limited to short-term exposure windows, we evaluated soy intake across multiple life stages. The sample comprised a large, population-based cohort of premenopausal women aged 35–45 years, free of conditions affecting soy consumption and not using exogenous hormones.

We conducted a comprehensive life course assessment of soy intake at ages 6–12, 13–18, 20–34, and during the past year using a validated soy FFQ that captures >90% of dietary isoflavone intake and incorporates isoflavone content based on locally sampled soy foods. Early-life recall was assisted by a life history calendar administered by trained interviewers. Mammographic percent density was measured objectively with a computer-assisted program and assessed by a single, experienced rater blinded to participant identity and exposures. The observed MD distributions aligned with previously reported values for Singapore Chinese premenopausal women [23]. Moreover, there was adequate within-population variation in soy intake, allowing for meaningful assessment of associations with MD across a wide range of exposure levels.

## 7. Conclusions

Our results indicate that the timing of soy exposure may impact adult breast tissue composition and could contribute to a reduced risk of breast cancer. These findings raise the possibility that breast tissue may be susceptible to dietary modulation during specific developmental windows and warrant further investigations.

## Figures and Tables

**Table 1:** Characteristics of study population (*N* = 815).

Characteristics	*N*	%
**Marital status**		
Married	668	82.0
Single	84	10.3
Divorced/separated	54	6.6
Widowed	9	1.1
**Education level**		
Primary or below	85	10.4
Secondary	222	27.2
Completed secondary	322	39.5
Post secondary—no degree	54	6.6
Post secondary—degree	131	16.1
Missing	1	0.1
**Work status**		
Homemakers	245	30.1
Part time/volunteer work	154	18.9
Full time	414	50.8
Missing	2	0.2
**Smoking habits**		
Non-smoker	736	90.3
Ex-smoker	45	5.5
Current smoker	34	4.2
**Drinking habits**		
Non-drinker	275	33.7
Occasional drinker	494	60.6
Current drinker	46	5.7
**Frequency of soy food intake**		
<1/week	82	10.1
1–2/week	293	36.0
3–4/week	186	22.7
5–6/week	101	12.4
>=7/week	153	18.8
**Soy protein intake (g/d)**		
Q1 (≤3.55)	203	24.9
Q2 (3.56–7.22)	204	25.0
Q3 (7.23–13.83)	204	25.0
Q4 (≥13.84)	204	25.0
**Soy isoflavone intake (mg/day)**		
Q1 (≤7.55)	203	24.9
Q2 (7.56–15.76)	204	25.0
Q3 (15.77–30.24)	204	25.0
Q4 (≥30.25)	204	25.0
	Mean ± SD *	Range
**Age (years)**	40.9 ± 2.94	35–45
**Body measurements**		
Body weight (kg)	56.5 ± 8.57	39.1–95.6
Body height (cm)	156.2 ± 5.26	142.5–173.2
BMI (kg/m^2^)	23.1 ± 3.32	16.4–37.5
Waist circumference (cm)	75.4 ± 8.10	58.5–110.8
**Reproductive variables**		
Age at menarche (years) (*N* = 799)	13.0 ± 1.65	9.0–19.0
Age at first birth (years) (*N* = 663)	27.2 ± 4.50	16.0–42.0
Number of live birth (*N* = 659)	1.85 ± 0.75	1–5
Duration of OC use (months) (*N* = 478)	40.10 ± 53.17	0.06–269.0
**Physical activity index**	23.0 ± 2.81	14.8–30.7
**Dietary intake**		
Energy intake (Kcal/day)	1666.7 ± 658.16	381.1–3906
Fat intake (g/day)	70.2 ± 41.84	6.1–472.8
**Mammographic density (%)**	38.8 ± 9.86	4.7–73.1

* SD = standard deviation.

**Table 2:** Soy protein (g/d) and soy isoflavone intake (mg/d) at different life stages [median, mean and standard deviation (SD)] (*N* = 815).

	Median	Mean	SD
	Soy protein (g/day)		
Age 6–12 y (T_3_)	6.60	9.33	9.62
Age 13–18 y (T_2_)	8.53	11.25	10.55
Age 20–34 y (T_1_)	9.48	12.57	10.74
Current (T_0_)	7.24	10.25	9.99
Mean of intake (T_0_, T_1_, T_2_, T_3_)	8.73	10.85	8.23
Mean of intake (T_2_ and T_3_)	7.97	10.29	9.57
Mean of intake (T_1_, T_2_ and T_3_)	8.68	11.05	9.05
	Soy isoflavones (mg/day)		
Age 6–12 y (T_3_)	15.38	21.35	21.49
Age 13–18 y (T_2_)	19.01	25.08	23.38
Age 20–34 y (T_1_)	20.38	27.15	23.13
Current (T_0_)	15.79	22.06	21.29
Mean of intake (T_0_, T_1_, T_2_, T_3_)	19.16	24.08	18.11
Mean of intake (T_2_ and T_3_)	18.29	23.38	21.35
Mean of intake (T_1_, T_2_ and T_3_)	19.43	24.69	20.08

**Table 3:** Correlation of dietary soy protein and isoflavone intake at different life stages (*n* = 815).

	T_0_	T_1_	T_2_	T_3_
	**Soy protein**			
Current (T_0_)	1.00	0.498	0.359	0.327
Age 20–34 y (T_1_)	0.498	1.000	0.642	0.535
Age 13–18 y (T_2_)	0.359	0.642	1.000	0.799
Age 6–12 y (T_3_)	0.327	0.535	0.799	1.000
	**Soy isoflavones**			
Current (T_0_)	1.000	0.508	0.359	0.324
Age 20–34 y (T_1_)	0.508	1.000	0.652	0.554
Age 13–18 y (T_2_)	0.359	0.652	1.000	0.807
Age 6–12 y (T_3_)	0.324	0.554	0.807	1.000

*p* < 0.001 for all correlations.

**Table 4:** Regression of soy protein/soy isoflavone intake at different life stages on MD after adjusting for confounders.

	β	SE	*p*-Value
**Soy protein intake (g/day)**			
Age 6–12 y (T_3_)	−0.071	0.032	0.028
Age 13–18 y (T_2_)	−0.067	0.029	0.023
Age 20–34 y (T_1_)	−0.052	0.029	0.075
Current (T_0_)	−0.003	0.032	0.93
Age 6–18 y (mean of T_2_ & T_3_)	−0.077	0.032	0.018
Age 6–34 y (mean of T_1_, T_2_ & T_3_)	−0.082	0.034	0.017
Life course intake (mean of T_0_, T_1_, T_2_, T_3_)	−0.079	0.039	0.041
**Soy isoflavone intake (mg/day)**			
Age 6–12 y (T_3_)	−0.034	0.014	0.018
Age 13–18 y (T_2_)	−0.030	0.013	0.024
Age 20–34 y (T_1_)	−0.023	0.014	0.090
Current (T_0_)	−0.001	0.015	0.960
Age 6–18 y (mean of T_2_ & T_3_)	−0.036	0.014	0.012
Age 6–34 y (mean of T_1_, T_2_ & T_3_)	−0.038	0.016	0.014
Life course intake (mean of T_0_, T_1_, T_2_, T_3_)	−0.037	0.018	0.034

Adjusted for: age, waist circumference, height, and number of live births.

**Table 5:** (**a**). Mammographic density (mean, SE) by soy protein intake by life stages and intake categories ^†^. (**b**) Mammographic density (mean, SE) by soy isoflavone intake by life stages and intake categories ^†^.

**(a)**				
	**Adjusted Mean ^†^**	**S.E.**	**95% CI**	**MD % Difference** **(High vs. Low Intake)**
Soy protein (T_0_)				
^a^ Low (*N* = 200)	38.59	0.60	37.42–39.76	
Moderate (*N* = 403)	39.27	0.42	38.45–40.09	
High (*N* = 202)	38.03	0.59	36.87–39.20	−1.47
*p*-Value	0.219			
Soy protein (T_1_)				
^a^ Low (*N* = 200)	39.29	0.60	38.12–40.47	
Moderate (*N* = 405)	38.80	0.42	37.98–39.63	
High (*N* = 200)	38.26	0.60	37.08–39.44	−2.69
*p*-Value	0.478			
Soy protein (T_2_)				
^a^ Low (*N* = 199)	39.70	0.60	38.53–40.88	
Moderate (*N* = 407)	38.97	0.42	38.14–39.79	
High (*N* = 199)	37.51	0.60	36.33–38.69	−5.84
*p*-Value	0.030			
Soy protein (T_3_)				
^a^ Low (Q1) (*N* = 200)	40.03	0.60	38.86–41.21	
Moderate (Q2 & Q3) (*N* = 405)	38.74	0.42	37.92–39.57	
High (Q4) (*N* = 200)	37.64	0.60	36.47–38.81	−6.35
*p*-Value	0.020			
Soy protein (T_2_, T_3_)				
^b^ Low (*N* = 226)	39.96	0.56	38.86–41.07	
Moderate (*N* = 354)	38.76	0.45	37.88–39.64	
High (*N* = 225)	37.66	0.56	36.56–38.77	−6.11
*p*-Value	0.015			
Soy protein (T_1_, T_2_, T_3_)				
^c^ Low (*N* = 161)	39.76	0.67	38.46–41.07	
Moderate (*N* = 491)	38.89	0.38	38.14–39.64	
High (*N* = 153)	37.44	0.68	36.10–38.78	−6.20
*p*-Value	0.048			
Soy protein (T_0_ to T_4_)				
^d^ Low (*N* = 154)	38.90	0.68	37.56–40.24	
Moderate (*N* = 482)	39.02	0.39	38.27–39.78	
High (*N* = 169)	38.03	0.65	36.76–39.31	−2.29
*p*-Value	0.420			
**(b)**				
	**Adjusted Mean ^†^**	**S.E.**	**95% CI**	**MD % Difference** **(High vs. Low Intake)**
Soy isoflavone (T_0_)				
^a^ Low (*N* = 200)	38.63	0.60	37.46–39.81	
Moderate (*N* = 403)	39.19	0.42	38.37–40.02	
High (*N* = 202)	38.14	0.59	36.97–39.31	−1.28
*p*-Value	0.334			
Soy isoflavone (T_1_)				
^a^ Low (*N* = 200)	39.25	0.60	38.08–40.43	
Moderate (*N* = 405)	38.82	0.42	37.99–39.64	
High (*N* = 200)	38.28	0.60	37.10–39.46	−2.53
*p*-Value	0.518			
Soy isoflavone (T_2_)				
^a^ Low (*N* = 198)	39.78	0.60	38.60–40.95	
Moderate (*N* = 408)	38.91	0.42	38.09–39.73	
High (*N* = 199)	37.56	0.60	36.38–38.74	−5.91
*p*-Value	0.031			
Soy isoflavone (T_3_)				
^a^ Low (*N* = 200)	39.96	0.60	38.80–41.14	
Moderate *(N* = 405)	38.84	0.42	38.02–39.66	
High (*N* = 200)	37.52	0.59	36.36–38.70	−6.5
*p*-Value	0.016			
Soy isoflavone (T_2_, T_3_)				
^b^ Low (*N* = 223)	39.85	0.57	38.74–41.00	
Moderate (*N* = 351)	38.84	0.45	37.96–39.73	
High (*N* = 231)	37.69	0.59	36.59–38.77	−5.73
*p*-Value	0.024			
Soy isoflavone (T_1_, T_2_, T_3_)				
^c^ Low (*N* = 158)	39.92	0.67	38.60–41.24	
Moderate (*N* = 488)	38.86	0.38	38.11–39.61	
High (*N* = 159)	37.46	0.67	36.14–38.78	−6.57
*p*-Value	0.033			
Soy isoflavone (T_0_ to T_4_)				
^d^ Low (*N* = 159)	39.19	0.68	37.86–40.52	
Moderate (*N* = 482)	38.99	0.39	38.23–39.75	
High (*N* = 174)	37.87	0.65	36.60–39.14	−3.73
*p*-Value	0.265			

SE—standard error, CI—confidence interval. ^†^ Least-square means and *p*-values from multivariate GLM, adjusted for age, waist circumference, height, number of livebirths, physical activity index. Score: Quartile (Q) belongs to Q1 = 1, Q2 = 2, Q3 = 3, Q4 = 4. ^a^ Low = 1, moderate = 2–3, high = 4. ^b^ Low = 2–3, moderate = 4–6, high = 7–8. ^c^ Low = 3–4, moderate = 5–10, high = 11–12. ^d^ Low = 4–6, moderate = 7–13, high = 14–16.

## Data Availability

The data presented in this study are not publicly available because they are part of an ongoing research project. Data may be obtained from the corresponding author upon reasonable request.

## Abbreviations

The following abbreviations are used in this manuscript:

MD	Mammographic density
SFFQ	Soy food frequency questionnaire
LHC	Life history calendar
SD	Standard deviation
ER	Estrogen receptor
GREB1	Gene regulated by estrogen in breast cancer 1
